# Stirring the POTS: Finding Symptom Patterns in Long COVID

**DOI:** 10.1016/j.jacadv.2025.101869

**Published:** 2025-08-27

**Authors:** Atsushi Mizuno

**Affiliations:** Department of Cardiovascular Medicine, St. Luke’s International Hospital, Tokyo, Japan

**Keywords:** long COVID, patient reported outcome, POTS, symptom

**Figure:**
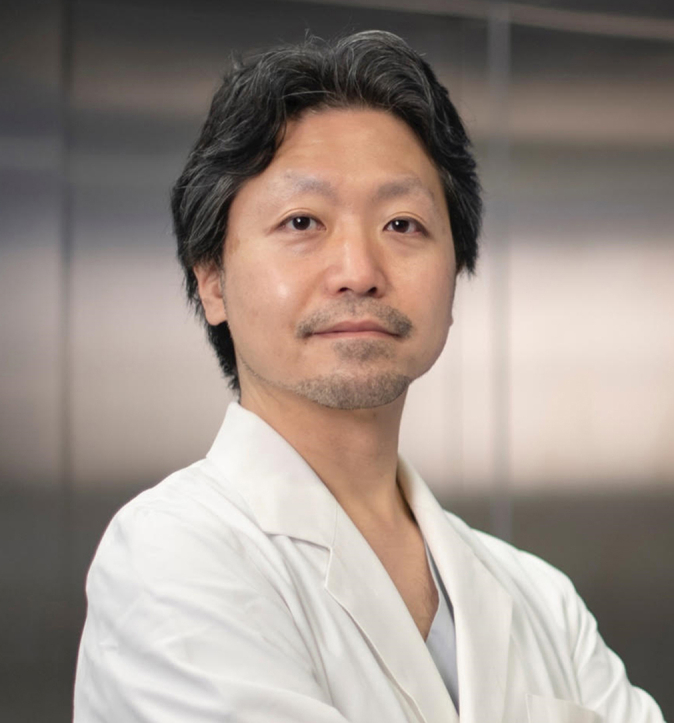

## Definition of long COVID

The prevalence of Long COVID varies widely depending on definitions and measurement methods. Some studies have suggested that 40% to 50% of individuals may experience persistent symptoms lasting more than 3 months after SARS-CoV-2 infection.^1^ The U.S. National Academies of Sciences, Engineering, and Medicine defines Long COVID is an infection-associated chronic condition that occurs after SARS-CoV-2 infection and is present for at least 3 months as a continuous, relapsing and remitting, or progressive disease state that affects one or more organ systems, which notably using the term “disease state” rather than “syndrome.” This deliberate choice reflects an effort to underscore the legitimacy and seriousness of the condition and to counteract the phenomenon of “medical gaslighting,” wherein patients’ symptoms are downplayed or dismissed due to their vague or poorly understood nature.^2^ Additionally, there are 2 particularly notable aspects of this new definition. First, a diagnosis of Long COVID can be made based solely on clinical course, without requiring documentation of prior infection. This reflects the realities of the pandemic—limited testing capacity early on, variable test sensitivity, and a decrease in testing rates in the later phases. Second, the definition is explicitly designed to be dynamic, with the expectation that it will evolve as further evidence emerges. This underlines the importance of continuous data collection from diverse clinical and patient-reported sources.

## Postural orthostatic tachycardia syndrome in the context of long COVID

Postural orthostatic tachycardia syndrome (POTS), a condition marked by tachycardia and a constellation of autonomic symptoms upon standing, has drawn increasing attention in the context of Long COVID. Originally characterized in 1993 by Philip Low at the Mayo Clinic, POTS is now widely recognized as one of the manifestations of orthostatic intolerance.^3^ POTS is defined as a clinical “syndrome” that is usually characterized by:

1) Frequent symptoms that occur with standing such as lightheadedness, palpitations, tremulousness, generalized weakness, blurred vision, exercise intolerance, and fatigue
2) An increase in heart rate of ≥30 beats/min when moving from a recumbent to a standing position held for more than 30 seconds (or ≥40 beats/min in individuals 12-19 years of age)
3) The absence of orthostatic hypotension (OH) (>20 mm Hg drop in systolic blood pressure). Typically defined by the following criteria:

Although POTS and OH are both classified under orthostatic intolerance, they differ in their clinical profiles—POTS tends to affect younger individuals and is associated with considerable psychological and functional burden, whereas OH is more common among older adults, those with diabetes, or individuals taking diuretics. POTS has also been linked to conditions such as myalgic encephalomyelitis/chronic fatigue syndrome. POTS is typically subtyped into hypovolemic, neuropathic, and hyperadrenergic forms, which often overlap. Understanding these subtypes requires a broader perspective on autonomic dysfunction (dysautonomia). From a cardiology perspective, particularly among electrophysiologists, POTS has garnered interest due to its clinical overlap with inappropriate sinus tachycardia. This resemblance prompted the Heart Rhythm Society to address POTS in its 2015 expert consensus statement, which included a discussion of diagnostic criteria and potential treatment strategies.^4^ At that time, invasive interventions such as sinus node modification and sinus node–sparing ablation were not recommended, reflecting concerns regarding safety and efficacy. However, with ongoing advancements in mapping and ablation technologies, these strategies are once again being explored in carefully selected cases.^5^ POTS, therefore, offers valuable insights into a range of domains—its symptoms are consistently triggered by a defined physical stimulus (orthostasis), its objective correlates are observable in vital signs, and its pathophysiology straddles autonomic dysfunction and, increasingly, abnormalities of the cardiac conduction system. As such, it serves as a clinically rich and mechanistically complex condition. In recent years, numerous cases of POTS have been reported under the diagnosis of Long COVID, highlighting its growing relevance in this emerging context.

## Symptom clustering and the role of pots in understanding long COVID

Mouslmani et al demonstrate that among individuals with Long COVID, those who report a diagnosis of POTS exhibit a greater clustering of symptoms by using The Yale LISTEN study.^6^ The Yale LISTEN study is a digital, decentralized, and participant-centric research initiative that recruits participants from Hugo Health Kindred, an online patient community composed of individuals interested in pandemic-related research. This community facilitates information exchange and enables members to contribute survey responses and health data to support research efforts. In this study, self-reported diagnoses were used to identify both Long-COVID and POTS status. While this approach may lack clinical validation in terms of formal diagnostic testing, interpreting such data as “Doctor-diagnosed conditions” provides a meaningful lens. In general, diagnoses related to COVID-19 tend to be underrepresented in administrative or clinical records. Conversely, self-report surveys can effectively capture patients with disabling symptoms who remain undiagnosed within the health care system. Such approaches increase diagnostic sensitivity, and in conditions like POTS—where diagnostic criteria are relatively well defined—they may also offer reasonable specificity. Nevertheless, it is essential to interpret the findings with caution, as the diagnoses reported in this study are not confirmed through standardized diagnostic procedures. Recognizing this limitation is critical when considering the implications of the data.^7^ Future comparisons with data from specialized autonomic clinics using validated questionnaires may yield valuable insights.

## From symptoms to clinical targets: challenges and future directions

The question of how clinicians and researchers should engage with subjective symptoms is once again at the forefront. As highlighted in the 2024 National Academies of Sciences, Engineering, and Medicine definition, persistent symptoms alone are sufficient to meet the diagnostic criteria for Long COVID, without the need for alternative diagnoses to be excluded or for laboratory-confirmed SARS-CoV-2 infection. This marks a significant departure from traditional diagnostic paradigms. Although the importance of objective indicators in diagnosis and treatment remains undiminished, Long COVID forces us to confront the complexity of symptoms that may not align neatly with conventional organ-based classifications. Considering this manuscript, POTS represents a rare anchor—a condition characterized by both well-defined subjective symptoms and measurable physiological changes (ie, orthostatic heart rate increase). While POTS does not impact survival, it significantly affects quality of life and has attracted growing interest as a target for therapeutic intervention. This study suggests a potential association between the broader symptomatology of Long COVID and the symptom clustering observed in POTS. Although causality remains unproven, treatment strategies anchored in objective indicators—such as those used in POTS—may yield downstream benefits across a wider array of symptoms. Taken together, these observations suggest that POTS could emerge as a potentially high-yield therapeutic target within the Long COVID spectrum.

Historically, POTS has been referred to by many names—such as “soldier’s heart” or “effort syndrome”—and has often been relegated to the periphery of medical recognition, earning the moniker “the Cinderella of medicine.” With Long COVID emerging as a patient-driven clinical construct, we may now have an opportunity to reevaluate such “conditions” with renewed legitimacy and scientific rigor. As we move toward identifying discrete syndromes and disease states within broader conditions like Long COVID, it becomes increasingly important to incorporate patient-participatory research and real-world data. Ultimately, such efforts will not only inform updates to Long COVID definitions but also establish a precedent for more inclusive and responsive clinical science.

## Funding support and author disclosures

Dr Mizuno has served as an Associate Editor for *JACC: Advances*.
